# Effects of Epidemic Diseases on the Distribution of Bonobos

**DOI:** 10.1371/journal.pone.0051112

**Published:** 2012-12-12

**Authors:** Bila-Isia Inogwabini, Nigel Leader-Williams

**Affiliations:** Durrell Institute for Conservation and Ecology, University of Kent, Canterbury, United Kingdom; University of Pittsburgh Center for Vaccine Research, United States of America

## Abstract

This study examined how outbreaks and the occurrence of Anthrax, Ebola, Monkeypox and Trypanosomiasis may differentially affect the distribution of bonobos (*Pan paniscus*). Using a combination of mapping, Jaccard overlapping coefficients and binary regressions, the study determined how each disease correlated with the extent of occurrence of, and the areas occupied by, bonobos. Anthrax has only been reported to occur outside the range of bonobos and so was not considered further. Ebola, Monkeypox and Trypanosomiasis were each reported within the area of occupancy of bonobos. Their respective overlap coefficients were: J = 0.10; Q_α = 0.05_ = 2.00 (odds ratios = 0.0001, 95% CI = 0.0057; Z = −19.41, significant) for Ebola; J = 1.00; Q_α = 0.05_ = 24.0 (odds ratios = 1.504, 95% CI = 0.5066–2.6122) for Monkeypox; and, J = 0.33; Q_α = 0.05_ = 11.5 (Z = 1.14, significant) for Trypanosomiasis. There were significant relationships for the presence and absence of Monkeypox and Trypanosomiasis and the known extent of occurrence of bonobos, based on the equations y = 0.2368Ln(x)+0.8006 (R^2^ = 0.9772) and y = −0.2942Ln(x)+0.7155 (R^2^ = 0.698), respectively. The positive relationship suggested that bonobos tolerated the presence of Monkeypox. In contrast, the significant negative coefficient suggested that bonobos were absent in areas where Trypanosomiasis is endemic. Our results suggest that large rivers may have prevented Ebola from spreading into the range of bonobos. Meanwhile, Trypanosomiasis has been recorded among humans within the area of occurrence of bonobos, and appears the most important disease in shaping the area of occupancy of bonobos within their overall extent of occupancy.

## Introduction

Conservation biologists have often underestimated the threat that infectious diseases pose to wildlife [1]. However, infectious diseases are now recognised to have a significant impact on some populations of wildlife. In the late 1990's, the concept of pathogen pollution was introduced to conceptualise the role that parasites play among the human-mediated threats to biodiversity [2]–[3]. Although many infectious agents are species-specific, a number of pathogenic organisms can cross the species barrier and cause severe clinical diseases in new hosts. For example, zoonotic diseases cross the barriers between humans and wildlife, and provide examples of bi-directional disease transmission whose impact can be severe and unpredictable. Thus a mild pathogen in one species may cross natural species barriers and emerge as a new infectious disease whose impacts are can be severe [4]–[5]. Therefore, understanding the role of infectious diseases in determining the current distribution and abundance of wildlife is of key importance for conservation. In this context, it is critical to further understand the role played by emerging infectious diseases and zoonoses transmitted between humans and great apes, whose close genetic relationship makes it more likely that they might ‘share’ many diseases.

Despite the close genetic relationship, only a few previous studies have sought to link disease occurrence and outbreaks in humans with the distributions of great ape populations. Western lowland gorillas are very sensitive to epidemic haemorrhagic diseases such as Ebola [6]–[12]. Indeed, recent Ebola epidemic outbreaks have decimated populations of western lowland gorillas in western Congo, as a result of which large expanses of suitable forest habitat currently remain empty, which in turn will influence plans for the future conservation of gorillas in the western Congo Basin. Mountain gorillas are susceptible to human respiratory diseases such as Influenza and Parainfluenza. Homsy [13] reviewed tourism regulations in light of epidemiological data and the risk of disease transmission between people and gorillas based on studies of captive gorillas, and showed that gorillas are susceptible to contracting human diseases, to which they lack the same resistance as humans. As a result, human pathogens, particularly those of respiratory diseases (such as measles, pneumonia), herpes and enteric diseases (such as polio, salmonella), can affect gorillas. Homsy [13] concluded that exposure to diseases makes tourism one of the single greatest threats to mountain gorilla survival.

Bonobos are the only species of great ape to occur within a single national jurisdiction, that of the Democratic Republic of Congo (DRC). The known extent of occurrence of bonobos is limited to an area encompassed by a large bend in the Congo River to the north, east and west, and by the Kwa-Lukenie-Kasai river system to the south. However, bonobos are patchily distributed within their known extent of occurrence and are known to occupy at least five separate areas that appear isolated from each other [14]–[22]. Furthermore, bonobos occupy some of these separate areas in significant numbers where there are continua of suitable habitats [22]–[23]. The patchy distribution of bonobos has so far been explained through various competing hypotheses, including: the topography and the history of land use; hunting by humans [24]; the presence of *Marantaceae* forests; and, the effects of diseases such as sleeping sickness [18]. However, none of these hypotheses fully explain the bonobo's patchy distribution, which seems to be influenced by a range of variables at different sites. Consequently, further research is needed to explain possible relationships between diseases and the distributions of great ape populations [25]–[26], especially for species such as bonobos where little is known about how disease might limit their distribution [18]. Therefore, this study aims to investigate the potential impacts of diseases in explaining the restricted yet patchy patterns of distribution of bonobos. This paper investigates: (1) the patterns of distribution of four diseases that have been documented or hypothesized to affect other species of great apes, and of other primates, and for which spatially explicit data were available on disease outbreaks. No such data could be located for Poliomyelitis in the Congo Basin. Consequently, the diseases examined comprise: Anthrax [8], [25]–[27], Ebola [6]–[12], Monkey pox [28] and Trypanosomiasis [18], for which we compare their patterns of distribution within the Congo Basin with the known distributions of bonobos. (2) The possible impacts of different diseases on the known extent of occurrence of bonobos, and/or on their known areas of occupancy within this extent of occurrence.

## Data and Methods

### Distribution of disease

Data on the distributions of outbreaks and occurrences of Anthrax, Ebola, Monkeypox and Trypanosomiasis, were mapped from document searches for clinically described, and serologically confirmed cases of each disease. Despite extensive searches of potential data sources, we were unable to locate any spatially explicit data on the distribution of Poliomyelitis within the extent of occurrence of bonobos, and so omitted this disease from our analysis. Data on the distribution of Anthrax were derived from Levine et al [29]. Data on the distribution of Ebola were derived from a combination of sources, principally IRCS [30], Walsh et al [31] and Peterson et al [32]. Data on the distribution of Monkeypox were derived from Ellis' map [28], combined with records generated by Levine et al [29]. When combined, these two data sets proved complementary and provided geo-referenced maps of Monkeypox distribution that could be easily overlaid on spatial data describing bonobo distribution. Spatially explicit data on the distribution of Trypanosomiasis only covered the extent of occurrence of bonobos. Therefore, we used only villages indicated by medical reports to constitute zones where Trypanosomiasis appeared to be endemic [33]. Data on bonobo distribution were derived from the Great Apes Survival Project (GRASP), in combination with the work of Fenart & Deblock [34].

### Data analysis

First, we calculated the geographical overlap between each of the four diseases and the known extent of occurrence of bonobos, using simple percentages of how many villages from which any disease was reported fell within the known extent of occurrence of bonobos.

Second, we created a binary data matrix on the presence and absence of bonobos, and plotted this against the presence of the four diseases to see if disease presence corresponded to areas occupied or unoccupied by bonobos over their known extent of occurrence. The presence or absence of bonobos was first compared with the known distributions of each disease, using the Jaccard J overlap measure as an indication of potential impact of each disease on the overall distribution of bonobos. A score of J = 1 means that bonobos live within the known distributions each disease, while a score of J = 0 implies that bonobos are totally absent from known distributions of each disease. Therefore, the closer the J-value is to 1, the more bonobos would seem to tolerate, or not be susceptible to, each disease.

Third, we used the Cochran Q-test on dual data sets to evaluate the relationship between the presence of declared disease foci and the absence of bonobos within their overall extent of occurrence. The Cochran Q-test has the formula:
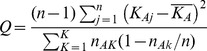
where K_Aj_ is the number of geographic locations for which ‘0’ was recorded by study j, 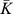
_A_ is the mean number of localities for which ‘0’ was recorded, K is the total number of localities, n is the number of studies and n_Ak_ is the number of studies that registered ‘0’ for locality k. Q has an approximately X^2^ distribution with n −1 degrees of freedom [35].

Because of the nature of the data, the Q-test can only assess the extent of the overlap between bonobo distribution and diseases that largely overlapped with the extent of occurrence of bonobos. Therefore, the Z-test was used to compare sequential contingencies using the log odds ratio. Given a series of binary data (0, 1), odds ratio **τ** is the ratio of number of subjects with the event in a group (1) relative to the number of subjects without the event (0). Log-odds ratio is the natural log of the odds ratio (β = ln (**τ**)), and the Z-value is obtained by the formula:
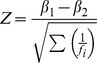
Wherein β_1_ represents the odds ratio for localities where outbreaks of diseases were confirmed in the country and β_2_ is the odds ratio of the localities within the bonobo range. ƒ*_i_* is the frequency in the *i* ^th^ cell and Z is the standard normal variate for N (0, 1). β is defined as a logistic model using the binary regression equation [36]:
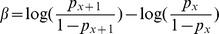
which, by the rules operating for logarithmic functions, can be simplified as:
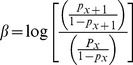
and whose plot indicate the fit of the data to the logistical model and gives the level of significance of inferred presence (or absence). Plotted and modeled β were generated using GENSTAT 5, a computer package that is robust in data analysis and easily operates generalized binary modes.

## Results

**Figure 1:**
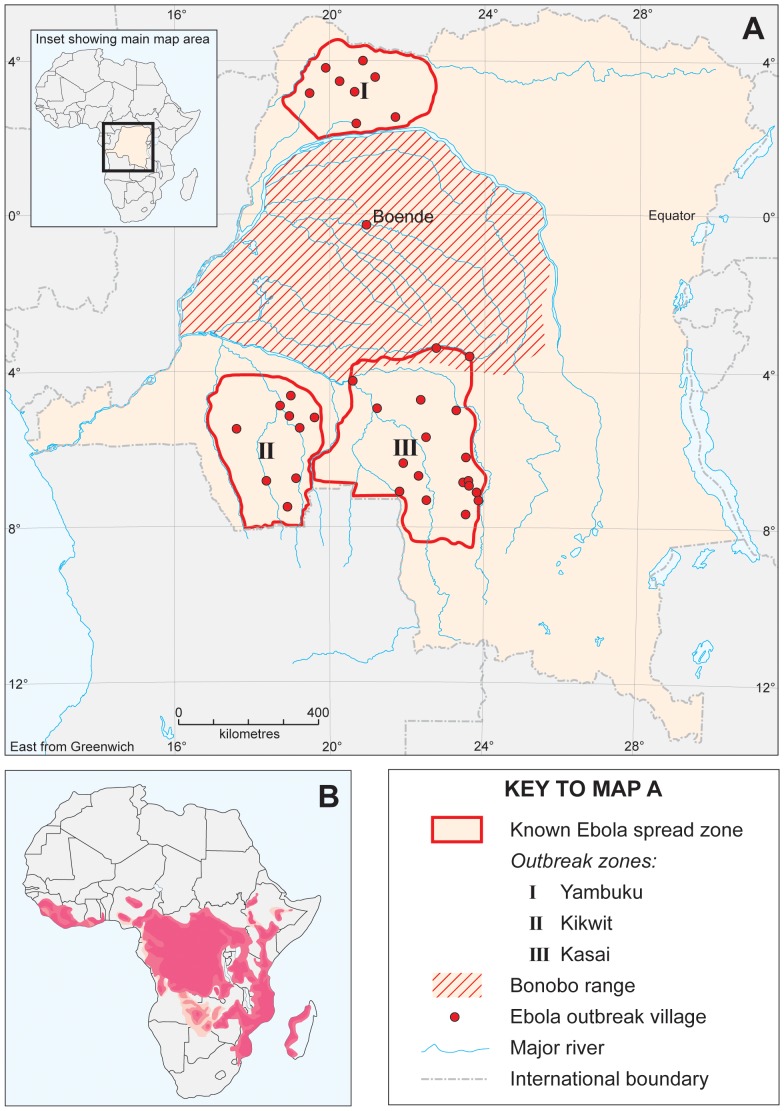
Distribution of bonobos relative to the known occurrence of Ebola within the Democratic Republic of Congo. Inset shows the known distribution of Ebola across Africa. A = Actual outbreak zone and occurrence points for Ebola, adapted from International Federation of Red Cross and Red Crescent Societies, 2009 (Walsh *et al.*, 2005). B = Modeled potential zone of Ebola outbreaks across Africa (after Peterson *et al.*, 2004). Grey shading in the main map represents the extent of occurrence of bonobos.

**Figure 2:**
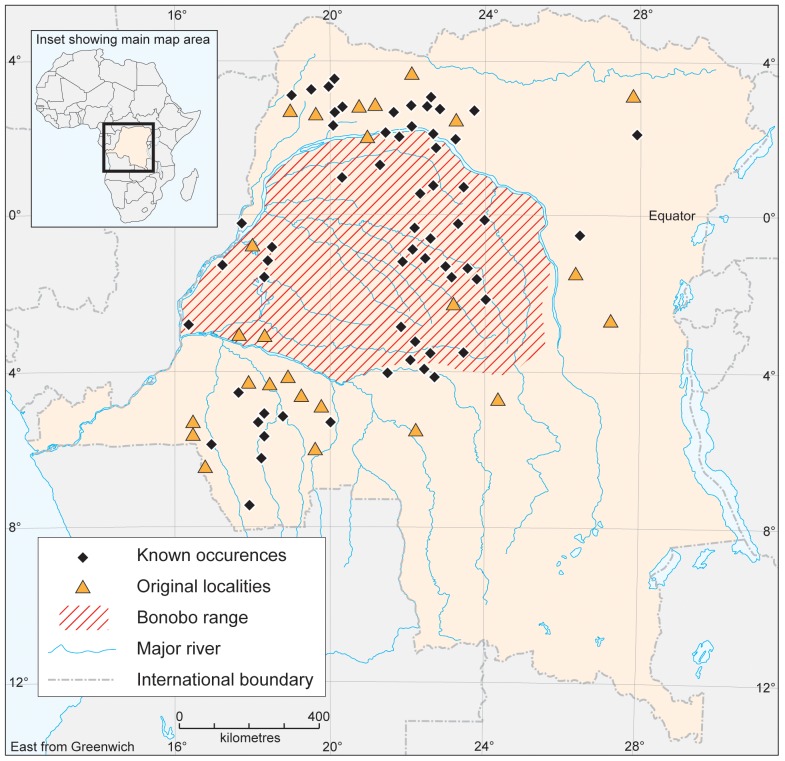
Distribution of bonobos and known occurrences of Monkeypox within DRC.

**Figure 3:**
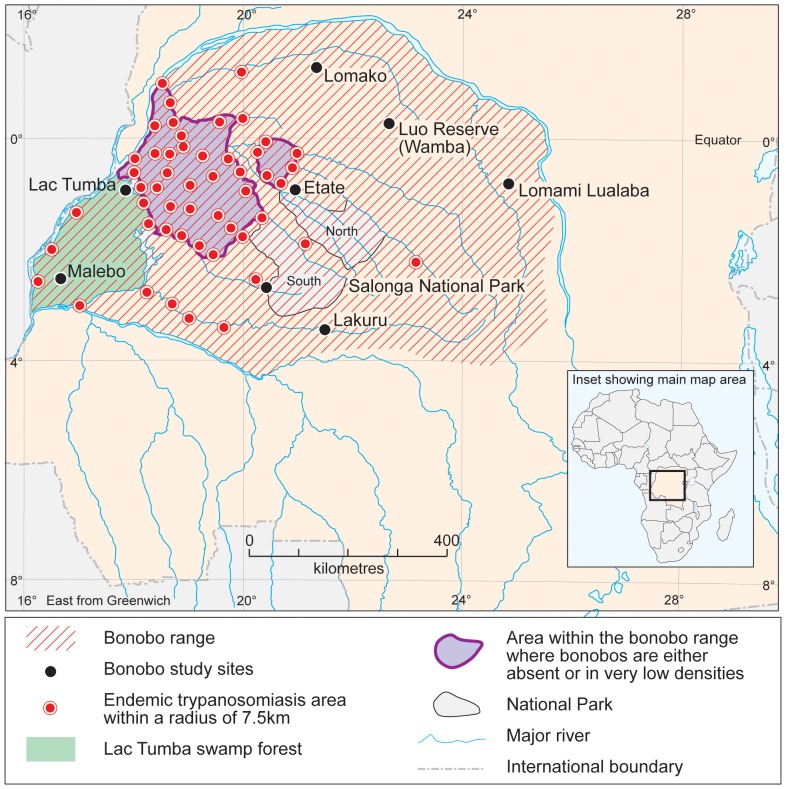
Extent of occurrence of bonobos and the occurrence of endemic Trypanosomiasis.

Only one outbreak of Anthrax has been reported within DRC (Table 1). This outbreak occurred *ca.* 400 km in a straight-line distance from the known extent of occurrence of bonobos, so Anthrax is not considered further in this analysis. In contrast, Ebola (J = 0.10; Q_α = 0.05_ = 2.00: Table 1) has been confirmed within the known extent of occurrence of bonobos, but only on three isolated occasions (Figure 1). One outbreak occurred at Boende, an area located at the centre of the known extent of occurrence of bonobos (Table 1). In further contrast, Monkeypox (J = 1.00; Q_α = 0.05_ = 24.0: Table 1) appears very widespread across the known extent of occurrence of bonobos (Figure 2). Likewise, Trypanosomiasis (J = 0.33; Q_α = 0.05_ = 11.5: Table 1) appears very widespread across the known extent of occurrence of bonobos (Figure 3), but discriminates itself from the areas occupied by bonobos (Table 1).

**Table 1:** The known occurrence of four diseases within the extent of occurrence and areas occupied by bonobos.

Disease	Country^1^	Range^2^	Overlap	J-value	Q_α = 0.05_	Z-value
Anthrax	1	0	-	-	-	-
Ebola	75	3	4.0%	0.10	2.00*	−19.41*
Monkeypox	62	38	61.9%	1.00	24.0^NS^	1.91^NS^
Trypanosomiasis	154	71	46.1%	0.33	11.5*	1.14*

1 Number of localities where the disease has been reported in DRC.

2 Number of localities where the disease has been confirmed in the bonobo range.

* Values that are <_α = 0.05_.

NS Values that are >_α = 0.05_.

**Figure 4:**
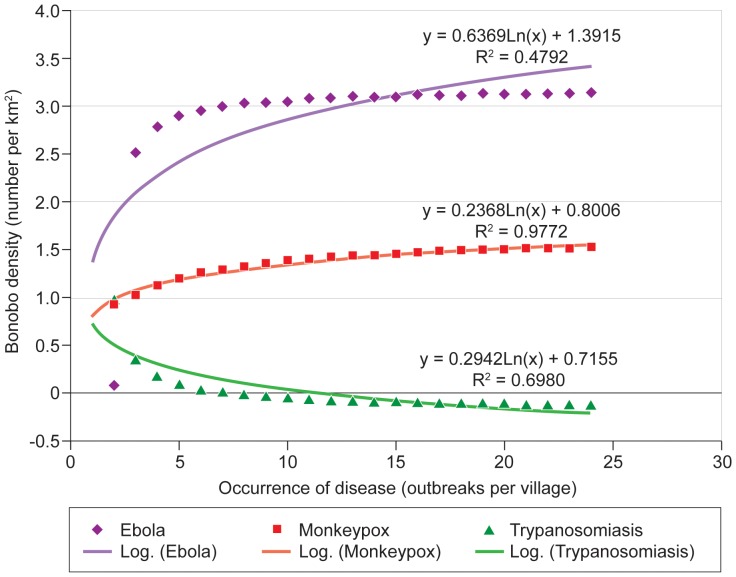
Binary regression models comparing disease occurrences and the distribution of bonobos across the Democratic Republic of Congo.

For Ebola, odds ratios were 0.0001 with 95% CI = 0.0057, while for Monkeypox, odds ratios were 1.504 with 95% CI = 0.5066–2.6122 (Table 1). Z-values (Table 1) were significant only for Ebola (−19.41) and Trypanosomiasis (1.14) indicating that disease patterns differed across areas occupied by bonobos. Thus patterns of diseases were either characterized by sporadic and unpredictable outbreaks, as in the case of Ebola, or high levels of endemism, as in the case of Trypanosomiasis. Quite strikingly, Monkeypox seems to be absent from areas within the extent of occurrence of bonobos where bonobos are absent (Figure 2). The J-value for Trypanosomiasis was 0.33, indicating that bonobos only occupied 33% sites where sleeping sickness was reported to be endemic. Figure 4 indicates only two areas where Trypanosomiasis appeared to be strongly endemic. Covering an area of ∼49,870 km^2^ out of the 562,040 km^2^ estimated for the whole extent of occurrence of bonobos, these two zones represent about 9% of the know extent of occurrence of bonobos.

The binary logistic regressions produced contrasting results (Figure 4). There was a positive but weak relationship (y = 0.6369Ln(x)+1.3915 (R^2^ = 0.4792) between the presence and absence of Ebola and the known extent of occurrence of bonobos. There was a significant but positive relationship (y = 0.2368Ln(x)+0.8006; R^2^ = 0.9772) for the presence and absence of Monkeypox and the known extent of occurrence of bonobos. This positive relationship suggests that bonobos can tolerate the presence of Monkeypox, which does not occur where there are no bonobos. There was a significant negative relationship (y = −0.2942Ln(x)+0.7155; R^2^ = 0.698) between the presence and absence of Trypanosomiasis and the areas of occupancy of bonobos. This negative coefficient suggests that bonobos are absent in areas where Trypanosomiasis is endemic, while present in areas where this disease is not endemic.

## Discussion

This is the first study to examine how outbreaks and occurrences of several diseases may differentially affect the distribution of a non-human species of great ape. By examining several diseases, we have been able to determine how each may differentially correlate with the extent of occurrence of, and the areas occupied by, bonobos. Unfortunately, we were unable to locate any spatially explicit data on the distribution of Poliomyelitis outbreaks within the bonobo range, and so had to omit this disease from further consideration. Nevertheless, our analysis for four diseases suggests four possibly distinct patterns. First, the only known occurrence of Anthrax in DRC was very far away from the known extent of occurrence of bonobos. Therefore, no inferences can be drawn regarding its possible effects on the known extent of occurrence of bonobos. Second, outbreaks of the Ebola occur regularly and quite close to the known extent of occurrence of bonobos. However, these outbreaks do not overlap with the known distribution of bonobos, suggesting that the extent of occurrence of bonobos may in part be explained by outbreaks of Ebola. Third and fourth, cases of both Monkeypox and Trypanosomiasis occur within the known extent of occurrence of bonobos. However, in the case of Monkeypox, there is a positive relationship between the occurrence of the disease and the areas occupied by bonobos. In contrast, in the case of Trypanosomiasis, the endemic occurrence of the disease shows a negative relationship with the areas occupied by bonobos. We now elaborate further on how each disease may affect the distribution of bonobos.

Anthrax is a potentially dangerous disease for great apes, having seriously affected chimpanzees in Dja, Cameroon [26]. However, searches to determine occurrences of Anthrax in Central Africa through the web and through health institutions provide few geographically referenced cases. Within DRC, the only spatially referenced case is the recent discovery of Anthrax in the carcasses of dead hippos (*Hippopotamus amphibius*) in the Lake Edward – Kasenyi region [37]. This single confirmed case was so far outside the known range of bonobos that no meaningful analysis of the potential impact of Anthrax on bonobos is possible (Table 1). However, this should not be taken to imply that Anthrax does not pose a potential threat to bonobos. Instead, wildlife managers should remain vigilant for possible outbreaks of Anthrax, and for its possible effects on great apes.

Outbreaks of the Ebola pandemic have almost exclusively remained outside the known range of bonobos (Figure 1), with three exceptions. Three cases of Ebola were reported in 2008 at Boende, located at the heart of the extent of occurrence of bonobos (Figure 1) and two other cases were reported to the very south of the extent of occurrence of bonobos, during the Ebola Kasai outbreak in 2007. These two localities at Dekese and Kole [30] are separated from the south of the core bonobo range by the Lukenie River. The weak positive correlation between Ebola and the extent of occurrence of bonobos (Figure 4) may simply be an artifact picturing the paucity of localities with value 1 (presence of Ebola). The same relationship may also be explained by including Boende in the spatial analysis, given it lies at the centre of the extent of occurrence on bonobos (Figure 1). Thus, the positive slope in the binary regression equation should not be interpreted as indicating that bonobos tolerate hemorrhagic diseases such as Ebola, as intuitively suggested by its low level of significance (R^2^ = 0.4792). Despite the supposed outbreaks of Ebola hemorrhagic disease at Boende, Dekese and Kole, large rivers may have served as barriers that have kept the ravaging effects of Ebola at a safe distance from bonobos. Likewise, Walsh et al [31] have suggested that major rivers in Central Africa may have played a key role in containing Ebola, and preventing waves of the disease from emerging outside of its original foci. Even though major rivers may have buffered bonobos from the deadly spread of the Ebola up to now, the hemorrhagic episode at Boende gives little cause for optimism that they will continue to act as a buffer in the long term. Indeed, Peterson et al [32] have suggested that the distributional model for Ebola (Figure 1) suggests that almost the entire range of the bonobo encompasses ecological conditions that could allow an irruption of Ebola at any time. As people increase their on-land movements, Ebola virus could have impacts on bonobos that prove similar to those that have swept across the ranges of the western chimpanzee and western lowland gorilla.

Confirmed cases of Monkeypox have overlapped extensively with the known extent of occurrence of bonobos (Figure 2). Logistic regression analysis showed no significant difference for the extent of overlap between bonobos and Monkeypox (Table 1). Areas of high bonobo density such as Lake Tumba, Lomako and Wamba showed a strong positive relationship with the distribution of Monkeypox (Figure 2; Table 1). One of two explanations may underpin this positive relationship. First, bonobos may enjoy natural protection from Monkeypox, even though it is known to affect other diurnal primates such as *Allenopithecus nigrovirdis*, *Cercopithecus ascanius* and *Cercopithecus mona* [28] that share their ranges with bonobos. Second, bonobos may have adapted to the presence of Monkeypox, which they can currently survive without experiencing severe symptoms. Whichever explanation is correct, the extent of overlap of Monkeypox and bonobos requires further investigation.

A potentially compelling result is that known occurrences of Trypanosomiasis occur in many villages from which bonobos were absent [21]. Indeed, 46% of villages (N = 154) listed by medical institutions as areas with historical and current records of endemic Trypanosomiasis lack bonobos. Interestingly, bonobo researchers have long recognized that these areas show very few signs of occupation by bonobos. Even finer scale data collected from the Lake Tumba region show that the villages with endemic Trypanosomiasis located along the Loolo River correlated perfectly (100%) with the areas where bonobos were locally absent from forests that lie at a mean distance of 7.5±2.5 km from the river. This zone corresponds with a belt running south from Lake Tumba to Lake Maindombe (Figure 3), from which signs of bonobos have been lacking since research began in this region [21]–[24]–[38].

Bonobos appear to be absent from areas where Trypanosomiasis is endemic (Figure 3; Table 1). Furthermore, recent data from Lake Tumba and historical records from Kano [21] suggest that bonobo density and distribution may both have been affected over years by the presence of Trypanosomiasis. Although possibly not the only determinant of bonobo distribution, these results support Kortlandt's hypothesis [18] that Trypanosomiasis may have an influence on the areas occupied by bonobos within their wider extent of occurrence.

Our current understanding suggests that epidemic diseases such as Ebola have played a role in influencing the actual distributions of species of great apes [6]–[8]–[12]. However, only one serious outbreak of Ebola has been confirmed in the extent of occupancy of bonobos (Figure 4), while Ebola has been confirmed to occur rather sporadically in areas of occupancy of bonobos. Therefore, if any endemic disease may have influenced the distribution of bonobos, Trypanosomiasis appears the most likely of these diseases. Equally, it should be noted that we were unable to collect data to discriminate effects of other diseases from other potential diseases such as Malaria and Poliomyelitis, which also occur within the species' range. The study also did not collect serological data to confirm the presence of sleeping sickness in bonobos. However, captive bonobos do actually contract human diseases [39], and the occurrence of Trypanosomiasis in areas where bonobos are absent provides compelling evidence that this disease affects the distribution of bonobos. One illustrative case is the region of Bekongo in the Salonga National Park [40], where *Marantaceae* species favoured by bonobos as their main food are very abundant yet bonobos were absent between 1997 and 2005. However, Trypanosomiasis is endemic at Boangi, which lies within a 7.5 km radius of Bekongo. Therefore, the presence of Trypanosomiasis may explain why bonobos do not occur in that region.

Rivers are used as transport routes while bonobo distribution and density gradients are often described using distances from the nearest river. Our findings, combined with results of the study in the Lake Tumba Swamp forest [23]–[24], indicate two possible explanations of why rivers may in part determine gradients in bonobo distribution. First, this relationship may reflect the effects of major human transport routes on wildlife. Second, this relationship may reflect that the vector of Trypanosomiasis, the tsetse fly *Glossina* spp. is also a riparian species and occupies river flood basin where bonobos seem to be absent.

In conclusion, this paper has demonstrated some correlations between human transmitted diseases and the distribution of bonobos. Taking into account results published by Mugisha *et al* [41], Kaiser *et al* [42], Henderson [43] and Bender [44] from sanctuaries, zoos and wild habitats, further research on potential disease transmission between bonobos and human populations is required, particularly in regions where bonobos occur in areas adjacent to villages such as Wamba and Lake Tumba region. Furthermore, all managers thinking of promoting ecotourism as a conservation tool to generate funding should, as an imperative, implement the screening of wildlife and human diseases in their programmes, to ensure a long-term epidemiological surveillance that might allow immediate reaction should there be an outbreak of any kind of disease.
